# Exosomal long non-coding RNA MSTRG.292666.16 is associated with osimertinib (AZD9291) resistance in non-small cell lung cancer

**DOI:** 10.18632/aging.103119

**Published:** 2020-05-06

**Authors:** Qinfang Deng, Qiyu Fang, Boxiong Xie, Hui Sun, Yuchen Bao, Songwen Zhou

**Affiliations:** 1 Department of Oncology, Shanghai Pulmonary Hospital, Tongji University School of Medicine, Shanghai, China; 2 Department of Thoracic, Shanghai Pulmonary Hospital, Tongji University School of Medicine, Shanghai, China

**Keywords:** non-small cell lung cancer, exosomes, epidermal growth factor receptor, long non-coding RNAs, osimertinib resistance

## Abstract

Acquired resistance of osimertinib is encountered in clinic treatment of non-small cell lung cancer (NSCLC). However, the molecular mechanisms of osimertinib resistance are not fully revealed. This study aimed to investigate the roles of exosomes in delivering osimertinib resistance in NSCLC. Exosomes were successfully isolated. LncRNA sequencing identified a total of 123 differentially expressed lncRNAs, including 45 upregulated lncRNAs and 78 downregulated lncRNAs. The relative expression level of lncRNA MSTRG.292666.16 was significantly upregulated in osimertinib-resistant plasma, osimertinib-resistant H1975R cells and their derived exosomes, compared with those in osimertinib- sensitive plasma, H1975 cells and exosomes (*P* < 0.05). Besides, osimertinib-resistant exosomes could regulate gene expressions induced by osimertinib, including miRNA-21, miRNA-125b, *TGFβ*, *ARF6 and c-Kit.* Osimertinib-resistant exosomes could be taken up by osimertinib-sensitive H1975 cells and resulting in osimertinib-resistance *in vivo*. Knockdown of lncRNA MSTRG.292666.16 decreased osimertinib resistance of H1975R cells. Our results suggest that exosomal lncRNA MSTRG.292666.16 might be associated with osimertinib resistance in NSCLC.

## INTRODUCTION

Lung cancer leads to considerable deaths in both women and men worldwide [1, 2]. According to the cancer statistics in 2018, more than two million of lung cancer cases were estimated to be diagnosed and 1.8 million cases were estimated to dead globally [1]. East Asia, especially China, is the region with highest incidence and mortality of lung cancer [3]. Non-small cell lung cancer (NSCLC) is the most common diagnosed lung cancer. Surgery, chemotherapy, radiotherapy are the traditional ways for treating lung cancer. Most patients in developing countries are diagnosed at the advanced stage of cancer development, and surgery is not enough to control progression of cancer. In last few years, epidermal growth factor receptor (EGFR) target therapy and immunotherapy have received increasing attention [4]. Especially, the success of EGFR tyrosine kinases inhibitors (TKIs) in treating lung cancer initiated the period of molecular-targeted cancer therapy [5].

Erlotinib and gefitinib are representative drugs of the first generation of EGFR-TKIs and are the first-line therapy for EGFR mutated NSCLC patients [6, 7]. Though most patients respond well at the beginning of treatment, almost all patients eventually developed acquired resistance mainly due to EGFR T790M mutation. The third generation of EGFR-TKI is developed to overcome T790M-mediated drug resistance and has shown encouraging effects [8, 9]. It also inhibits EGFR-sensitive mutations and is expected to become the first-line drug for treating NSCLC. Osimertinib is one of the third-generation EGFR-TKIs which could inhibit mutated EGFR alleles, including T790M, L858R, and Ex19del [10]. However, acquired resistance of osimertinib was independently reported by several studies [11–13]. The molecular mechanisms of patients developing acquired resistance of osimertinib were heterogeneous. Through mutation studies, EGFR C797S mutation, EGFR L718Q mutation, KRAS G12S mutation, CMET amplification and HER2 amplification were identified to be responsible for this acquired resistance [14–18]. Recently, Zhao et al. identified recurrent EGFR V834L mutation might also contribute to resistance mechanisms of osimertinib [19]. Besides, ERBB2, PIKECA, RB1 and KRAS were also reported to be involved in osimertinib resistance development [20]. However, the mechanisms of osimertinib resistance are not fully understood. With the wide application of osimertinib, elucidation of molecular mechanisms of osimertinib resistance and developing strategies for alleviating this resistance is important for improving NSCLC prognosis.

Exosomes are a type of extracellular vesicles with a size of 40 nm-150 nm [21]. They are present in almost all cell supernatants and human body fluids [22–27]. Exosomes are reported to be involved in cell-to-cell communication by transferring genetic information, including long non coding RNA (lncRNA), microRNA (miRNA), messenger RNA (mRNA), DNA and proteins [28–31]. In addition, cancer cell derived exosomes could be released to tumor microenvironment and modulate drug resistance of cancer cells [32–35].

Emerging evidences have reported that lncRNAs might participate in drug resistance and might serve as therapeutic targets [36], such as lncRNA RP11-828N2.4 [37], lncRNA SBF2-AS1 [38], lncARSR [39]. However, whether and which lncRNA derived from cancer-resistant cells confer osimertinib resistance to sensitive cells still needs to be investigated.

In this study, we isolated the exosomes from plasma of NSCLC patients before osimertinib treatment and their corresponding resistant plasma and characterized their lncRNA profile. Differentially expressed lncRNAs between osimertinib-resistance exosomes and osimertinib-sensitive exosomes were identified and functions of one critical lncRNA in transferring osimertinib resistance were further investigated *in vivo*. These results may aid in understanding the molecular mechanism of osimertinib resistance and help develop new therapy for osimertinib-resistant NSCLC patients.

## RESULTS

### The isolation and identification of plasma derived exosomes

**Figure 1:**
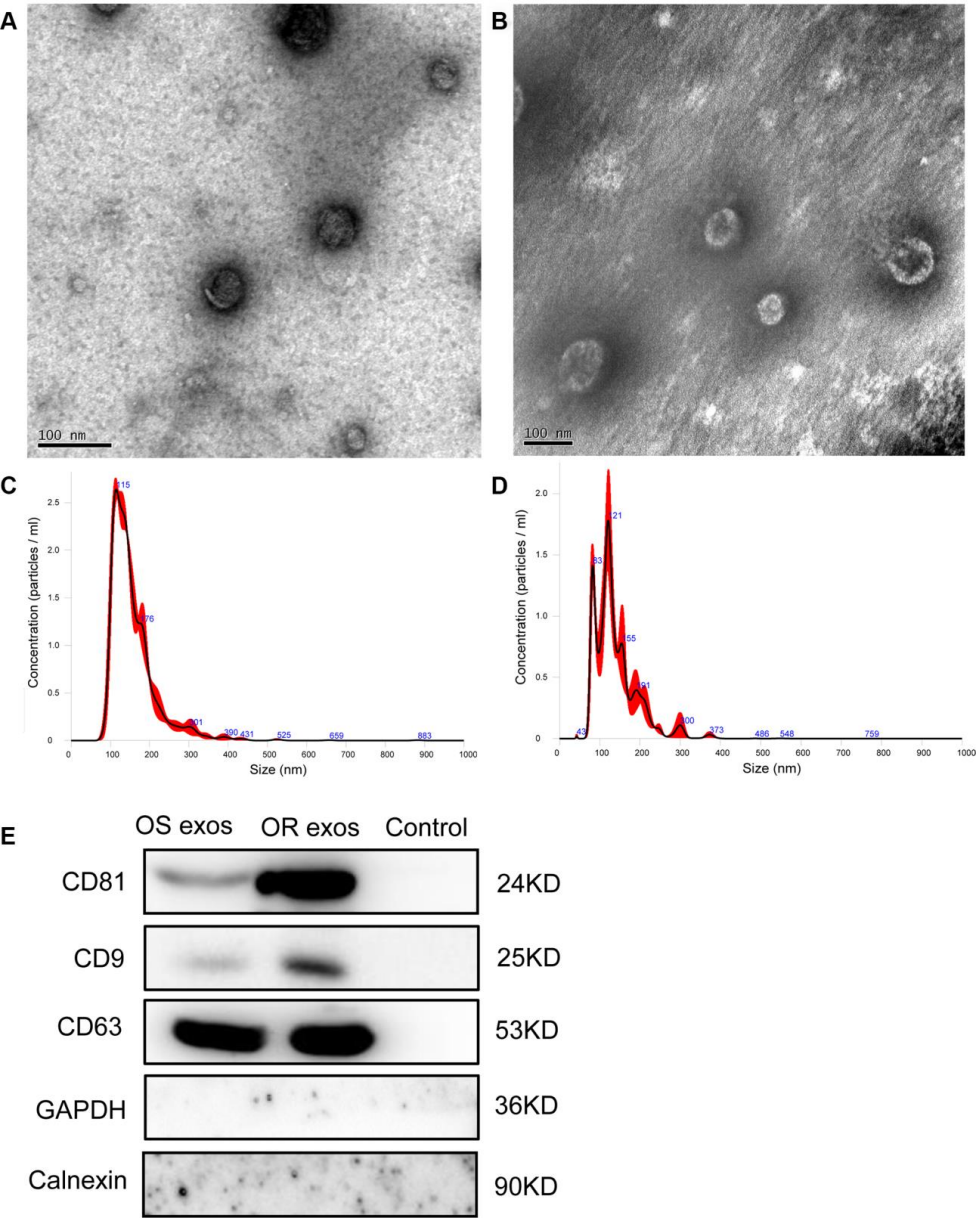
**Characterization of exosomes.** (**A**) Representative transmission electron microscopy (TEM) image of exosomes isolated from NSCLC patients before osimertinib treatment. Scale bar: 100 nm; (**B**) Representative TEM image of exosomes isolated from plasma of osimertinib-resistant NSCLC patients. Scale bar: 100 nm; (**C**) Nanoparticle tracking analysis of the size of exosomes isolated from NSCLC patients before osimertinib treatment; (**D**) Nanoparticle tracking analysis of the size of exosomes isolated from plasma of osimertinib-resistant NSCLC patients. (**E**) Western blot analysis of exosomal marker CD9, CD63 and CD81. Calnexin, which is an integral protein of the endoplasmic reticulum, and GAPDH were used as negative control for identification of exosomes. Supernatant was used as control samples. OS exos: Osimertinib-sensitive exosomes; OR exos: Osimertinib-resistant exosomes.

The exosomes from the plasma of NSCLC patients before osimertinib treatment and those of osimertinib-resistant patients were isolated by ultracentrifugation. To identify whether the isolated extracellular particles were exosomes or not, we performed TEM, NTA and western blot to characterize them. The representative morphology obtained by TEM revealed that the particles were round or oval-shaped vesicles with a double-layered membrane (Figure 1A). The size distribution of the purified particles was measured by NTA, which shows a peak diameter of 115 nm for osimertinib-sensitive exosomes and a peak diameter of 121 nm for osimertinib-resistant exosomes (Figure 1B). Besides, western blot analysis confirmed the protein expression of exosomal specific markers, CD9, CD63 and CD81 (Figure 1C). Taken together, these results suggested the extracellular particles isolated in plasma were exosomes.

### Characterization of the lncRNA profile of osimertinib-resistant plasma derived exosomes and osimertinib-sensitive plasma derived exosomes

**Figure 2:**
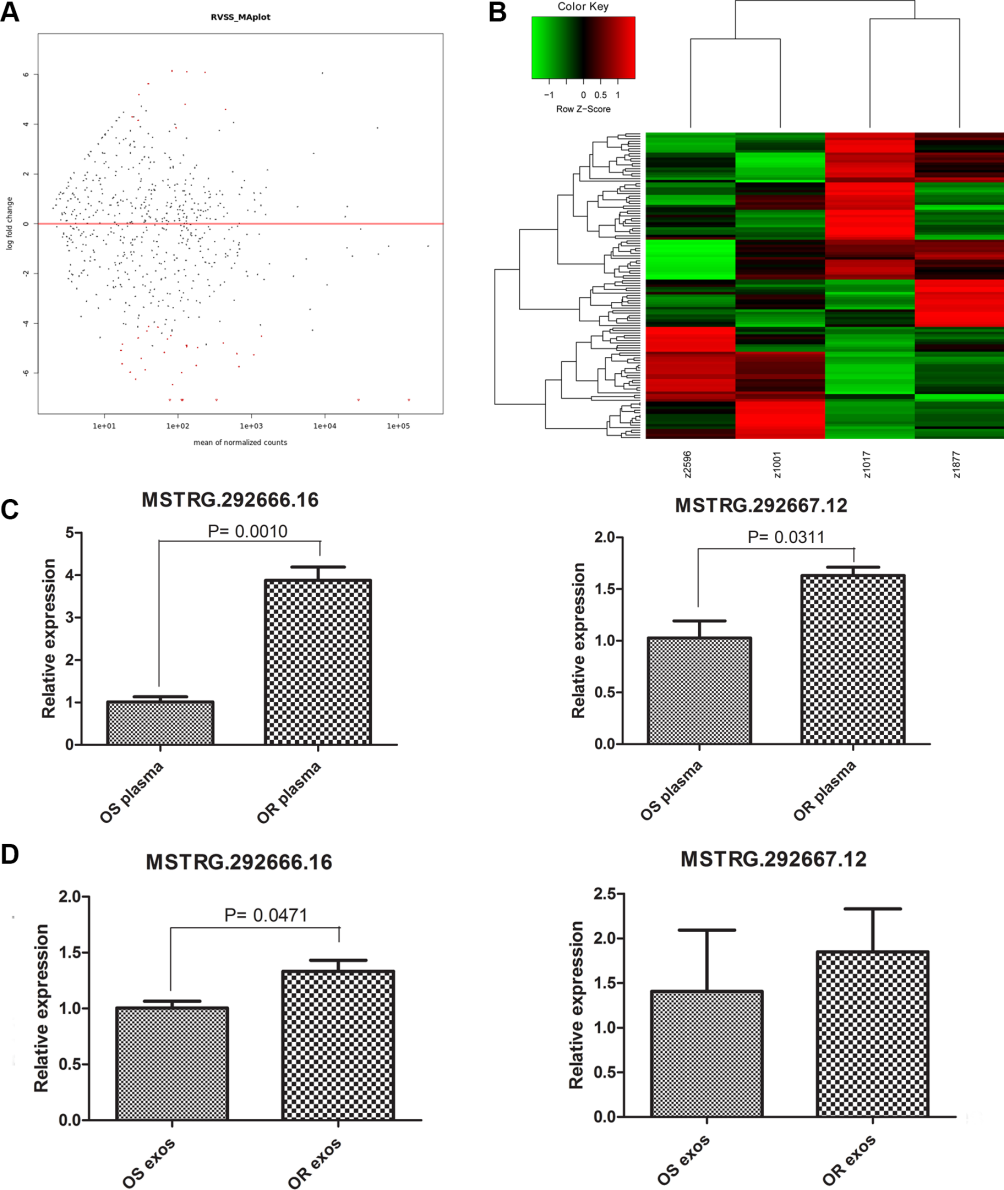
**Characterizing of long non-coding RNAs (lncRNAs) profiles.** (**A**) MA plot displayed the differentially expressed lncRNAs between osimertinib-resistant exosomes and osimertinib-sensitive exosomes. (**B**) Heatmap displayed the differentially expressed lncRNAs between osimertinib-resistant exosomes and osimertinib-sensitive exosomes. z2596 and z1001 are exosomes isolated from two patients before osimertinib treatment, while z1017 and z1877 are exosomes isolated from the same patients acquired osimertinib resistance. (**C**) qRT-PCR determined the relative expression of lncRNA MSTRG.292666.16 and lncRNA MSTRG.292667.12 between osimertinib-resistant plasma and osimertinib-sensitive plasma. OS: osimertinib-sensitive; OR: osimertinib-resistant. (**D**) qRT-PCR determined the relative expression of lncRNA MSTRG.292666.16 and lncRNA MSTRG.292667.12 between osimertinib-resistant exosomes and osimertinib-sensitive exosomes. OS: osimertinib-sensitive; OR: osimertinib-resistant.

LncRNA might be transferred by exosomes and contributed to drug resistance. Therefore, we further investigated the lncRNAs that might play important roles in osimertinib resistance. LncRNA sequencing was performed for the osimertinib-resistant exosomes and osimertinib-sensitive exosomes. Based on the criteria of |log2FC | >1 and FDR < 0.05, a total of 123 differentially expressed lncRNA were identified, including 45 upregulated lncRNAs and 78 downregulated lncRNAs (Supplementary Table 1, Figure 2A, 2B). Two upregulated lncRNAs, MSTRG.292666.16 (log2FC = 6.05318, *P* = 0.009671) and MSTRG.292667.12 (log2FC = 3.853559, *P* =0.034019) were further verified by qRT-PCR in both plasma and exosomes. As shown in Figure 2C, the relative expression levels of MSTRG.292666.16 and MSTRG.292667.12 were all significantly upregulated in osimertinib-resistant plasma compared with those in osimertinib-sensitive plasma (*P* < 0.05). Further verification was conducted in osimertinib-resistant exosomes and osimertinib-sensitive exosomes. Results showed that the relative expression levels of MSTRG.292666.16 were significantly upregulated in osimertinib-resistant exosomes compared with those in osimertinib-sensitive exosomes (*P* < 0.05), which is in line with the lncRNA sequencing results. However, no significant difference between these two groups was detected for lncRNA MSTRG.292667.12 (*P* > 0.05, Figure 2D). Therefore, we chose the lncRNA MSTRG.292666.16 for further analysis.

### H1975R cell-derived exosomes could be taken up by H1975S cells

**Figure 3:**
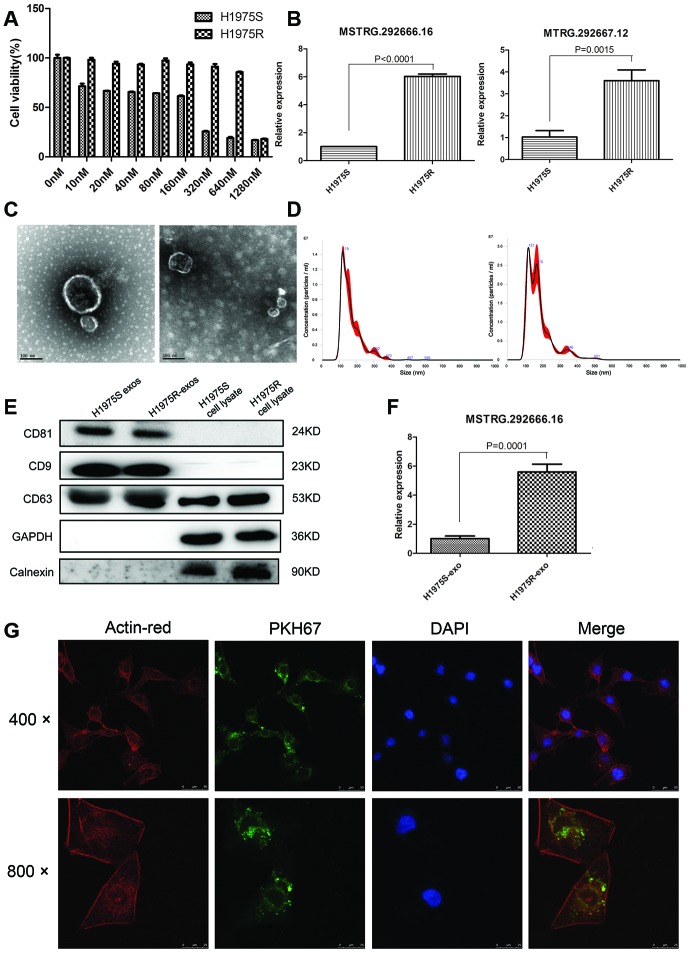
**Establishment of osimertinib-resistant H1975 cell lines.** (**A**) CCK-8 assay was conducted by treating H1975 cells with different concentrations of osimertinib. (**B**) Relative expression of lncRNA MSTRG.292666.16 and MSTRG.292667.12 in H1975 sensitive (H1975S) cells and H1975 resistant (H1975R) cells. (**C**) Representative TEM image of exosomes isolated from H1975S cells (left) and H1975R cells (right). Scale bar: 100 nm; (**D**) Nanoparticle tracking analysis of the size of exosomes isolated from H1975S cells (left) and H1975R cells (right). (**E**) Western blot analysis of exosomal marker CD9, CD63 and CD81. GAPDH and Calnexin were used as negative control. (**F**) Relative expression of lncRNA MSTRG.292666.16 in exosomes isolated from H1975S cells (H1975S-exo) and H1975R (H1975R-exo) cells. (**G**) The uptake of the PKH67 labelled osimertinib-resistant exosomes was evident in H1975 cells after 12 h of incubation. Cytoskeleton was dyed with actin-red, exosomes were dyed with PKH67. Scale bar, 10 μm.

Previous studies have demonstrated that exosomes may involve in drug resistance through transferring functional genetic materials. To confirm whether osimertinib resistance could be transferred by exosomes, an osimertinib-resistant H1975 cell line, H1975R, was established after 6 months of continuous exposure to stepwise- increasing concentrations of osimertinib. CCK-8 assay suggested that cell viability of H1975R cells was not significantly changed even being treated with 640 nM osimertinib (Figure 3A). qRT-PCR suggested that lncRNA MSTRG.292666.16 and MSTRG.292667.12 were significantly upregulated in H1975R cells compared with those in H1975S cells (*P* < 0.001, Figure 3B). Then, exosomes of H1975R and H1975S were isolated from the conditioned culture medium. TEM showed these exosomes were typical cup-shaped nanoparticles (Figure 3C). NTA suggested the peak diameter of H1975S-exosomes was 119 nm and that of H1975R-exosomes was 121 nm (Figure 3D). Western blot further confirmed the positive expression of exosomal markers CD9, CD63 and CD81 and negative expression of Calnexin (Figure 3E). These results suggested that the exosomes were successfully isolated. Besides, qRT-PCR suggested that expression of lncRNA MSTRG.292666.16 was significantly increased in H1975R-exosomes compared with that in H1975S-exosomes (*P* < 0.001, Figure 3F).

To investigate whether exosomes could be uptaken by recipient cells, H1975S cells were cocultured with PKH67- labeled H1975R exosomes. Examination using confocal microscopy showed that PKH67 green fluorescence signals were visible around the nuclei and in the cytoplasm of H1975S cells. This result confirmed the uptake of PKH67-labeled H1975R exosomes by H1975S cells (Figure 3G).

### Osimertinib resistant exosomes reverse osimertinib induced gene expression changes in H1975 cells

**Figure 4:**
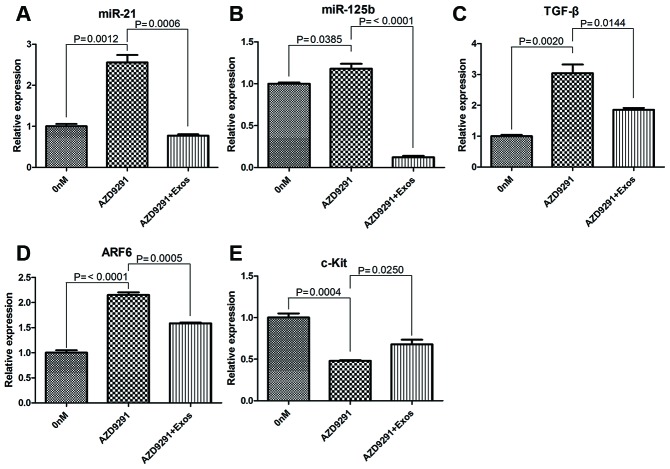
qRT-PCR determined the relative expression of miR-21 (**A**), miR-125b (**B**), *TGFβ* (**C**), *ARF6* (**D**) and *c-Kit* (**E**).

The effects of osimertinib-resistant exosomes on expression of several genes, including miR-21, miR-125b, *TGFβ*, *ARF6* and *c-Kit* were detected. The relative expression levels of miR-21, miR-125b, *TGFβ* and *ARF6* were significantly upregulated after treated with 100 nM osimertinib for 24 h. However, co-incubated with 10 μg/ml osimertinib-resistant exosomes could reverse the effects of osimertinib alone on gene expression changes (*P* < 0.05, Figure 4A–4D). The relative expression of *c-Kit* was significantly downregulated in osimertinib treated H1975 cells, while co-incubated with osimertinib-resistant exosomes could significantly upregulated the expression of *c-Kit* (*P* < 0.05, Figure 4E). These results suggested that osimertinib-resistant exosomes could regulate gene expressions induced by osimertinib.

### Osimertinib resistant exosomes induce the resistance of H1975 cells to osimertinib

**Figure 5:**
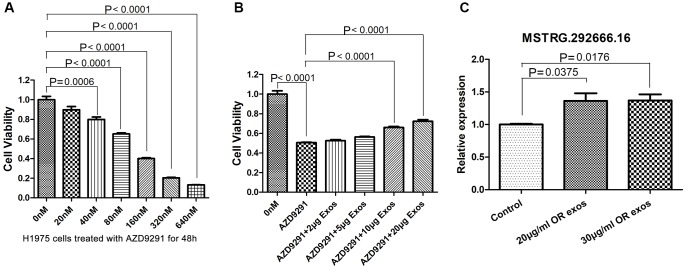
**Osimertinib-resistant exosomes induce the resistance of H1975 cells to osimertinib.** (**A**) Cell viability was assessed by CCK-8 assays. H1975 cells were treated with different concentrations of AZD9291 for 48h. (**B**) Cell viability was assessed by CCK-8 assays. H1975 cells were treated with AZD9291 and then incubated with different concentrations of osimertinib-resistant exosomes. (**C**) qRT-PCR determined the relative expression of lncRNA MSTRG.292666.16.

To further investigate the functional roles of osimertinib-resistant exosomes in the resistance of lung cancer cells to osimertinib, H1975 cells were treated with 100 nM osimertinib for 24 h and then incubated with different concentrations of osimertinib-resistant exosomes for 48 h. First, H1975 cells were treated with different concentrations of osimertinib to determine the treating concentration of osimertinib. As shown in Figure 5A, the cell viability of H1975 was significantly decreased in an osimertinib-dependent manner. The cell viability of H1975 cells treated with 40 nM and above osimertinib was significantly lower than that in control group (*P* = 0.0006). The IC50 was calculated and result showed that IC50 of osimertinib was 118.7 nM. Therefore, 118.7 nM osimertinib was used for further experiment.

In order to investigate whether osimertinib resistance could be transferred by exosomes, H1975 cells were co-cultured with 118.7 nM osimertinib and different concentrations of osimertinib-resistant exosomes. CCK8 assay showed that co-culture with osimertinib-resistant exosomes can significantly reduce the osimertinib sensitivity of H1975 cells and improve their relative survival rate in a dose-dependent manner (Figure 5B). Especially, the cell viability of H1975 cells incubated with 10 μg/ml and 20 μg/ml osimertinib-resistant exosomes was increased significantly compared with those without exosomes incubation (*P* < 0.0001).

We further investigated whether the significant upregulated lncRNA was also transferred to H1975 cells by exosomes. Relative expression of MSTRG.292666.16 in H1975 cells as well as in H1975 cells incubated with osimertinib-resistant exosomes were detected by qRT-PCR. Results showed that the relative expression of MSTRG.292666.16 in H1975 cells incubated with 20 μg/ml and 30 μg/ml osimertinib-resistant exosomes was significantly upregulated compared with that in H1975 cells without treatment (*P* < 0.05, Figure 5C).

### LncRNA MSTRG.2992666.16 might be associated with osimertinib resistance

**Figure 6:**
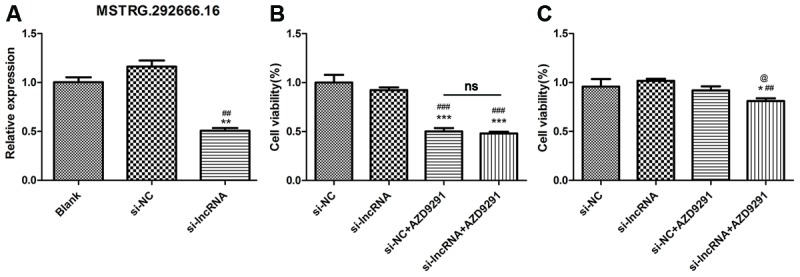
**LncRNA MSTRG.2992666.16 might be associated with osimertinib resistance.** (**A**) Relative expression of lncRNA MSTRG.292666.16 in blank, si-NC and si-lncRNA MSTRG.292666.16 group. ** indicates *P* < 0.01 compared with blank group; ## indicates *P* < 0.01 compared with siNC group. (**B**) CCK-8 assay of H1975S cells treating with AZD9291 and si-lncRNA MSTRG.292666.16. *** indicates *P* < 0.001 compared with si-NC group; ### indicates *P* < 0.001 compared with si-lncRNA group; ns indicates not significant. (**C**) CCK-8 assay of H1975R cells treating with AZD9291 and si-lncRNA MSTRG.292666.16. * indicates *P* < 0.05 compared with si-NC group; ## indicates *P* < 0.01 compared with si-lncRNA group; @ indicates P < 0.05 compared with siNC+AZD9291 group.

To further investigate the significance of MSTRG.292666.16 in osimertinib-resistance, we transferred siRNA-MSTRG.292666.16 into H1975 cells. As shown in Figure 6A, the relative expression of MSTRG.292666.16 in H1975 cells was significantly decreased (*P* < 0.01). CCK-8 assay suggested that knock down of MSTRG.292666.16 did not significantly affect cell viability of H1975S cells. However, cell viability of H1975R cells treated with siRNA-MSTRG.292666.16 was significantly decreased compared with siRNA-NC group (*P* < 0.05, Figure 6B). This result indicated that lncRNA MSTRG.292666.16 might be associated with osimertinib resistance.

## DISCUSSION

In this study, we identified the differentially expressed lncRNAs between exosomes derived from osimertinib-resistant plasma and those derived from osimertinib-sensitive plasma. Further, we investigated the functional role of a specific lncRNA with osimertinib resistance. Our data revealed that lncRNA MSTRG292666.16 was upregulated in osimertinib-resistant plasma derived exosomes. More importantly, we revealed that extracellular lncRNA MSTRG.292666.16 was transferred by exosomes, and exosomal lncRNA MSTRG292666.16 contribute to acquired osimertinib resistance of lung cancer cells.

Osimertinib has become widely used in many countries, and has shown promising response in NSCLC patients with T790M mutation [8, 9]. However, acquired resistance inevitably occurs. Therefore, understanding the mechanism of acquired osimertinib resistance in NSCLC is of great significance.

Exosomes play important role in cellular communication through transferring genetic information, including lncRNAs [40]. In this study, we performed lncRNA sequencing analysis to identify the DElncRNAs between osimertinib-resistant exosomes and the parental osimertinib-sensitive exosomes. We identified a total of 123 differentially expressed lncRNA, including 45 upregulated lncRNAs and 78 downregulated lncRNAs between these two groups and finally identified lncRNA MSTRG292666.16 as a potential lncRNA participating in osimertinib resistance. Moreover, we also demonstrated that this lncRNA can be incorporated into exosomes and contributed to acquired osimertinib resistance.

We further investigated whether lncRNA MSTRG292666.16 can be packaged into exosomes, and whether exosomal lncRNA MSTRG292666.16 could mediate osimertinib resistance. qRT-PCR results suggested the expression of lncRNA MSTRG292666.16 was detectable and markedly increased in the H1975 cells incubated with the extracted exosomes from osimertinib -resistant plasma. Treatment of the H1975 cells with exosomes containing lncRNA MSTRG292666.16 induced an enhanced osimertinib resistance. To conclude, lncRNA MSTRG292666.16 could be transferred by exosomes and participated in the development of osimertinib resistance.

Further, we checked the effects of osimertinib-resistant exosomes on gene expression of miR-21, miR-125b, TGFβ, ARF6 and c-Kit. These genes have been reported to be related with drug resistance previously. For example, miR-21 is involved in acquired resistance of EGFR-TKI by activating PI3K-Akt pathway and down-regulating PTEN and PDCD4.in NSCLC [41]. Besides, it was reported to mediate serveral cancers resistance to sorafenib and carmustine [42–44]. MiR-125b was reported to be related with drug resistance of cetuximab [45], doxorubicin [46], cisplatin, TNF-related apoptosis-inducing ligand [47]. Exosomal miR-125b was reported to be a predictive biomarker for mFOLFOX6-based chemotherapy in colorectal cancer patients [48]. Our results showed that the relative expression of miR-21, miR-125b, TGF*β* and *ARF6* were significantly upregulated after treated with osimertinib, while osimertinib-resistant exosomes could reverse these effects. Besides, the relative expression of *c-Kit* was significantly downregulated in osimertinib treated H1975 cells, while co-incubated with osimertinib-resistant exosomes could significantly upregulated the expression of *c-Kit.* These results demonstrated that osimertinib-resistance exosomes could regulate the gene expressions induced by osimertinib. However, the exact mechanism of exosomes in regulating these gene expressions is still warrant for investigation.

However, our study has some limitations. First, the sample size used for lncRNA sequencing is relatively small, because it’s hard to gather qualified samples. Second, deeper investigation on molecular mechanism of lncRNA MSTRG292666.16 in regulating osimertinib resistance should be further conducted in *in vitro* and in *in vivo* studies. Third, the role of lncRNA MSTRG292666.16 on osimertinib resistance was investigated in one cell line of H1975. Further investigations in other lung cancer cell lines are also warranted.

In conclusion, our study demonstrated that the exosome-mediated transfer of lncRNA MSTRG292666.16 might be associated with osimertinib resistance in NSCLC. LncRNA MSTRG292666.16 might be served as a therapeutic target to overcome osimertinib resistance.

## MATERIALS AND METHODS

### Patients

Two male NSCLC patients underwent osimertinib treatment from the department of oncology, Shanghai Pulmonary Hospital were included in this study. These patients were diagnosed with left upper-lobe adenocarcinoma with distal bone metastases. They were 56 and 57 years old respectively and both of them have smoking history. They were primarily given first-generation EGFR-TKIs. However, acquired resistance was developed. Genetic analysis of circulating tumor cells detected EGFR T790M mutation. Then, osimertinib was given and osimertinib acquired resistance was also developed after about 6 months treatment. The plasma before osimertinib treatment and their corresponding resistant plasma (each pair was from the same patient) were collected for exosome isolation.

### Exosome isolation

Exosomes were extracted from the plasma of patients before osimertinib treatment (sensitive group) and their corresponding resistant plasma (resistant group) through ultracentrifugation as reference described with few modifications [21]. Briefly, plasma was sequentially centrifuged at 4°C: 3,000 ×g for 15 min, and 12,000 ×g for 30 min to eliminate large cell debris. Then, the supernatant was collected and filtered by a 0.22 μm filters (Millipore, USA) to remove large particles, followed by ultracentrifugation at 4°C, 120,000 ×g, for 60 min. The supernatant was collected and then underwent another ultracentrifugation at 4°C, 120,000 ×g, for 60 min. The concentration of exosomes was determined by a pierce bicinchoninic acid (BCA) protein assay kit (Thermo Scientific, Waltham, MA, USA). Then, the exosomes were resuspended in phosphate-buffered saline (PBS) for further use.

Exosome-depleted fetal bovine serum (FBS) was prepared by ultracentrifugation at 100,000 ×g at 4°C for 16 h. The cells were transferred to 10% exosome-depleted FBS at 80% confluence and incubated for 48 h. The conditioned culture medium of H1975 and H3255 cells were collected and underwent differential centrifugation at 4°C: 500 ×g for 5 min, and 2000 ×g for 30 min. Then, the supernatant was mixed with pre-cooled 16% PEG6000 (v:v = 1:1, Sangon Biotech, Shanghai, China), and placed at 4°C overnight. The solution was centrifugated at 10,000 ×g for 60 min at 4°C. Then, 1 ml PBS was added to the supernatant and centrifugated 100,000 ×g for 70 min at 4°C. The exosomes were resuspended in PBS for further use.

### Characterization of exosomes

Transmission electron microscopy (TEM) was used to visualize exosomes as previously described [29]. Briefly, 10 μl fresh exosomes were fixed by 2% osmic acid at 4°C for 2h. Then, the fixed exosomes were dehydrated in gradient alcohol (50%, 70%, 80%, 90% and 100%), infiltrated in acetone and embedding medium (v: v = 2:1) and then embedded. The sections were dyed with uranyl acetate and lead acetate, and were captured by TEM (JEM-1010; JEOL, Tokyo, Japan).

The particle size of exosomes was determined by NanoSight NS300 equipped with a 450 nm laser (Malvern Panalytical, Malvern, UK) and analyzed by Nanoparticle Tracking Analysis (NTA) software (Malvern Panalytical).

### Western blot

The exosomes were lysed in radio-immunoprecipitation assay (RIPA) buffer (Beytime, Shanghai, China) supplemented with 1 mM PMSF. The lysate was centrifuged at 4°C, 12000 ×g for 10 min. Protein concentrations were evaluated by BCA kit. After being separated by SDS-PAGE, the proteins were transferred to polyvinylidene difluoride membranes (PVDF; Bio-Rad, USA). The primary antibodies, including anti-CD9 (1:1000; ab92726, Abcam), anti-CD63 (1:1000; A5271, ABclonal), anti-CD81 (1:1000; ab109201, Abcam), anti-calnexin (1:1000; 10427-2-AP, Proteintech) and anti-GAPDH (1:5000; 10494-1-AP, Proteintech) were added and incubated at 4°C overnight. After washing, the blots were incubated with the second antibodies (goat anti-rabbit IgG (H+L) -horseradish peroxidase (HRP), 1:1000 at 37°C for 2h. At last, the proteins were visualized by Millipore ECL system.

### Exosomal lncRNA next-generation sequencing

Exosomal RNA was extracted by miRNeasy serum/Plasma kit (QIAGEN, Waltham, MA). A total amount of 1.5 μg qualified RNA per sample was used to generate sequencing libraries using NEBNextR Ultra^TM^ Directional RNA Library Prep Kit for IlluminaR (NEB, USA). The libraries were sequenced on an Illumina Hiseq platform at Yanzai biotechnology Co. ltd. (Shanghai, China) to generate paired-end reads.

### Bioinformatics data analysis

Raw data of fastq format were preprocessed to obtain high quality clean data. The transcriptome was assembled using StringTie (version 1.3.1) based on the reference genome. LncRNAs were identified as described in reference [49]. The expression of lncRNA was calculated based on FPKMs by using StringTie (version 1.3.1). Differentially expressed lncRNAs (DElncRNAs) were identified using the DESeq R package (version 1.10.1) based on the criteria of |log2FC (fold change) | >1 and false discovery rate (FDR) < 0.05.

### Cell culture

Human lung cancer cell H1975 was purchased from the Chinese Academy of Sciences Cell Bank (Shanghai, China). Cells were maintained in DMEM containing 10% FBS, 1% penicillin and 1% streptomycin and incubated at 37°C and 5% CO_2_.

### Exosome labeling and tracking

Exosomes were labeled with the green-fluorescing PKH67 (Sigma Aldrich, St. Louis, MO, USA) as instructed by manufacture [50]. Briefly, 2 ml Diluent C mixed with 16 μl PKH67 were added to exosomes and incubated for 5 mins at 25 °C. Four milliliter 1% BSA was added to stop the reaction. Exosomes were washed once with PBS and then processed at 120,000 × g for 90 min at 4°C to remove excess PKH67.

The culture supernatant of lung cancer cell H1975 were discarded and replaced with DMEM supplemented with exosome-free FBS (Yanzai biotechnology, Shanghai, China). Labeled exosomes were cocultured with H1975 cells for 24 h and then fixed with 4% paraformaldehyde for 20 min. PBS containing 0.1% Triton X-100 was added to cells for 5 min and washed 3 times. 200 μL ActinRed (Cat. no.: KGMP0012, KeyGEN biotech, Nanjing, China) was then added and cells were incubated in dark for 30 min. At last, cells were stained with mounting medium containing DAPI. The uptake of labeled exosomes by H1975 cells was observed by a Leica laser scanning confocal microscope (TCS SP8, Leica, Wetzlar, Germany).

### Establishment of osimertinib-resistant H1975 cells

The osimertinib-resistant H1975 cells were established by continuous exposure to stepwise-increasing concentrations of osimertinib according to references [51, 52]. Briefly, H1975 cells of 70%-80% confluence were cultured in DMEM supplemented with a low concentration of osimertinib (10 nM) at the beginning for 72 h. Then, cells were cultured in drug-free medium to recover the survival cells. The selection cycles were continued with gradually increased concentrations of osimertinib from 10 nM to 1280 nM. Each concentration was repeated 3 cycles. H1975 cells became osimertinib-resistant after about 6 months. Viable cells were gathered and cultured in drug-free medium. The cell viability of H1975-resistant cells was tested by cell counting Kit-8 (CCK-8 assay). These osimertinib-resistant cells were denoted as H1975R cells. Their parental H1975 cells were propagated in drug-free medium and were denoted as H1975S cells.

### Transfection of lncRNA MSTRG.292666.16

siRNA of lncRNA MSTRG.292666.16 and its negative control (NC) were synthesized by Sangon Biotech (Shanghai, China). siRNA-MSTRG292666.16 and si-NC were transfected into H1975 cells by using lipofectamine2000 (Thermo, USA) according to previously described procedure [53].

### Cell viability assay

Lung cancer cells H1975S and H1975R were seeded in 96-well plate at 0.5 ×10^4^ cells per well and incubated with 100 nM osimertinib for 24 h. Then, cells were diluted in exosome-free FBS to final concentrations of 2 μg/ml, 5 μg/ml, 10 μg/ml and 20 μg/ml. Cell viability was detected with the CCK-8 assay (Beyotime, Shanghai, China) after 48 h and incubated for another 4 h. OD450 was measured on a microplate reader (MK3, Thermo Fisher, USA).

### RNA extraction and quantitative reverse transcription (qRT) -PCR assays

Extraction of total RNA from tissues and cultured cells was performed using Trizol (Invitrogen) and exosomal RNA was extracted by miRNeasy serum/Plasma kit (QIAGEN). RNA was reverse transcribed into complementary DNA using the PrimeScript RT reagent Kit (TaKara, Nanjing, China) and then RT-PCR analyses were performed with Gotaq® Green Master Mix (TaKara, Nanjing, China). The results were normalized with GAPDH. Primers for lncRNAs (MSTRG.292666.16 and MSTRG.292667.12) and internal controls were purchased from Ribobio (Guangzhou, China). Primers are shown in Supplementary Table 2. qRT-PCR was carried out on ABI 7500 real-time PCR system (Applied Biosystems, Foster City, CA, USA). Fold change was determined as 2^-ΔΔCt^ in gene expression.

### Statistical analysis

Molecular experiments were done in triplicate and data were shown as mean ± standard deviation. Difference between groups was detected by Student’s *t* test in Graphpad Prism 5.0. *P* < 0.05 was set as statistically significant level.

### Ethics statement

Investigation has been conducted in accordance with the ethical standards and according to the Declaration of Helsinki and according to national and international guidelines and has been approved by the authors' institutional review board.

## Supplementary Material

Supplementary Tables

Supplementary Table 1
