# Supplementary material for: Exosomal long non-coding RNA MSTRG.292666.16 is associated with osimertinib (AZD9291) resistance in non-small cell lung cancer
Source: Aging (Albany NY). 2020 May 6;12(9):8001–15. doi: 10.18632/aging.103119 (PMC7244069; doi:10.18632/aging.103119)
Supplement: Supplementary Tables [file aging-12-103119-s002..pdf]

SUPPLEMENTARY TABLES

Please browse Full Text version to see the data of Supplementary Table 1.

Supplementary Table 1. The list of differentially expressed lncRNAs.

Supplementary Table 2. Primers for quantitive real-time PCR.

Gene	Forward primer (5’-3’)	Reverse primer (5’-3’)
TGFβ	GGCCAGATCCTGTCCAAGC	GTGGGTTTCCACCATTAGCAC
cKit	CGTTCTGCTCCTACTGCTTCG	CCCACGCGGACTATTAAGTCT
ARF6	GGGAAGGTGCTATCCAAAATCTT	CACATCCCATACGTTGAACTTGA
MSTRG.292666.16	CTGGAGTGCAGTGGCTATTC	AGGCTGAGGTGGGAGGAT
MSTRG.292667.12	AGGCTGAGGTGGGAGGAT	CTGGGCAACATAGCGAGAC
hsa-miR-21-RT	GTCGTATCCAGTGCAGGGTCCGAGGTATTCGCACTGGATACGACTCAACA	
hsa-miR-21	ACACTCCAGCTGGGTAGCTTATCAGACTGAT	GTGCAGGGTCCGAGGT
hsa-miR-125b-RT	GTCGTATCCAGTGCAGGGTCCGAGGTATTCGCACTGGATACGACTCACAA	
hsa-miR-125b	GCGCTCCCTGAGACCCTAAC	GTGCAGGGTCCGAGGT
hsa-U6-RT	GTCGTATCCAGTGCAGGGTCCGAGGTATTCGCACTGGATACGACAAAATATG	
hsa-U6	CTCGCTTCGGCAGCACA	AACGCTTCACGAATTTGCGT
GAPDH	TGACAACTTTGGTATCGTGGAAGG	AGGCAGGGATGATGTTCTGGAGAG
