# Supplementary material for: Exosomal long non-coding RNA MSTRG.292666.16 is associated with osimertinib (AZD9291) resistance in non-small cell lung cancer
Source: Aging (Albany NY). 2020 May 6;12(9):8001–15. doi: 10.18632/aging.103119 (PMC7244069; doi:10.18632/aging.103119)
Supplement: Supplementary Table 1 [file aging-12-103119-s001..docx]

Supplementary Table 1. The list of differentially expressed lncRNAs.

| AccID | log2FC | P value | Style |
| --- | --- | --- | --- |
| MSTRG.1092194.12 | 2.755571889 | 0.039380712 | up |
| MSTRG.762571.3 | 2.845828089 | 0.047298831 | up |
| MSTRG.47827.5 | 3.06959229 | 0.028337395 | up |
| MSTRG.1005262.1 | 3.087930742 | 0.032254312 | up |
| MSTRG.315790.32 | 3.145714602 | 0.037704008 | up |
| MSTRG.358116.10 | 3.197537619 | 0.028114003 | up |
| MSTRG.411801.1 | 3.23559013 | 0.034335295 | up |
| MSTRG.693443.1 | 3.305763318 | 0.019009116 | up |
| MSTRG.205925.42 | 3.379600515 | 0.024167541 | up |
| MSTRG.383403.4 | 3.436785949 | 0.040510935 | up |
| MSTRG.299473.1 | 3.44075209 | 0.030466854 | up |
| MSTRG.693573.1 | 3.442860438 | 0.045626249 | up |
| MSTRG.1004843.48 | 3.455175433 | 0.046367431 | up |
| MSTRG.490260.1 | 3.475236012 | 0.020036901 | up |
| MSTRG.693310.3 | 3.476986128 | 0.043969155 | up |
| MSTRG.762446.1 | 3.5706977 | 0.039219354 | up |
| MSTRG.762785.7 | 3.591925167 | 0.044478653 | up |
| MSTRG.60081.1 | 3.605957047 | 0.038176352 | up |
| MSTRG.315790.6 | 3.625353826 | 0.011764757 | up |
| MSTRG.571758.1 | 3.717769579 | 0.035509713 | up |
| MSTRG.47972.1 | 3.739846278 | 0.011183425 | up |
| MSTRG.47791.77 | 3.745424669 | 0.018500641 | up |
| MSTRG.693438.6 | 3.745632371 | 0.025398502 | up |
| MSTRG.292667.12 | 3.853558806 | 0.034013689 | up |
| MSTRG.1092194.9 | 3.855743883 | 0.003891775 | up |
| MSTRG.762811.5 | 3.891572431 | 0.041478787 | up |
| MSTRG.47828.2 | 3.898383954 | 0.006307583 | up |
| MSTRG.442578.6 | 3.998392389 | 0.004655306 | up |
| MSTRG.357648.8 | 4.066625266 | 0.012245402 | up |
| MSTRG.108509.7 | 4.141608458 | 0.024178903 | up |
| MSTRG.762395.1 | 4.159624095 | 0.003557898 | up |
| MSTRG.571611.5 | 4.273648624 | 0.020683598 | up |
| MSTRG.108509.9 | 4.296297209 | 0.003821591 | up |
| MSTRG.442641.6 | 4.300161031 | 0.008038093 | up |
| MSTRG.490268.1 | 4.481255034 | 0.01604646 | up |
| MSTRG.47791.79 | 4.540562015 | 0.007162246 | up |
| MSTRG.357649.5 | 4.603938714 | 0.00098096 | up |
| MSTRG.490339.1 | 4.723118448 | 0.008754803 | up |
| MSTRG.762571.42 | 4.799662767 | 0.003653121 | up |
| MSTRG.693567.2 | 5.188521424 | 0.001717967 | up |
| MSTRG.1092199.2 | 5.62685897 | 0.001190286 | up |
| MSTRG.292666.16 | 6.053180102 | 0.009670861 | up |
| MSTRG.47791.50 | 6.088766623 | 2.06E-06 | up |
| MSTRG.47864.1 | 6.107638565 | 2.87E-05 | up |
| MSTRG.108515.5 | 6.142976338 | 0.000146934 | up |
| MSTRG.571609.1 | -2.46343 | 0.047607 | down |
| MSTRG.47790.84 | -2.58653 | 0.026874 | down |
| MSTRG.358115.12 | -2.62971 | 0.041188 | down |
| MSTRG.631909.18 | -2.68784 | 0.028084 | down |
| MSTRG.47791.85 | -2.71892 | 0.030298 | down |
| MSTRG.205924.19 | -2.75684 | 0.033714 | down |
| MSTRG.762571.28 | -2.80021 | 0.041402 | down |
| MSTRG.631909.30 | -2.90782 | 0.041438 | down |
| MSTRG.1092209.1 | -2.91012 | 0.048396 | down |
| MSTRG.762571.65 | -2.95269 | 0.015058 | down |
| MSTRG.47790.55 | -3.03625 | 0.026274 | down |
| MSTRG.138951.1 | -3.04513 | 0.024112 | down |
| MSTRG.762778.100 | -3.07601 | 0.034116 | down |
| MSTRG.631909.48 | -3.09589 | 0.023256 | down |
| MSTRG.47790.33 | -3.09685 | 0.031604 | down |
| MSTRG.631926.1 | -3.10756 | 0.040832 | down |
| MSTRG.631917.8 | -3.11567 | 0.023476 | down |
| MSTRG.1004843.47 | -3.17332 | 0.04617 | down |
| MSTRG.953368.1 | -3.17539 | 0.020502 | down |
| MSTRG.762778.13 | -3.18101 | 0.021989 | down |
| MSTRG.762723.2 | -3.28327 | 0.032172 | down |
| MSTRG.47791.88 | -3.3027 | 0.017366 | down |
| MSTRG.762778.61 | -3.35172 | 0.023866 | down |
| MSTRG.315790.25 | -3.36686 | 0.010356 | down |
| MSTRG.277532.1 | -3.38431 | 0.031969 | down |
| MSTRG.762778.71 | -3.50278 | 0.010896 | down |
| MSTRG.490449.1 | -3.53649 | 0.023695 | down |
| MSTRG.557571.30 | -3.67942 | 0.01365 | down |
| MSTRG.572936.1 | -3.72356 | 0.021081 | down |
| MSTRG.1004389.1 | -3.74314 | 0.016018 | down |
| MSTRG.108330.1 | -3.75982 | 0.020714 | down |
| MSTRG.762785.5 | -3.79358 | 0.010137 | down |
| MSTRG.442814.1 | -3.84833 | 0.022288 | down |
| MSTRG.1092199.1 | -3.88106 | 0.01943 | down |
| MSTRG.315790.1 | -3.92335 | 0.011842 | down |
| MSTRG.837401.2 | -3.95251 | 0.006809 | down |
| MSTRG.631814.1 | -4.00722 | 0.009291 | down |
| MSTRG.490209.3 | -4.02909 | 0.005332 | down |
| MSTRG.762553.1 | -4.02997 | 0.011976 | down |
| MSTRG.1092194.14 | -4.0648 | 0.019898 | down |
| MSTRG.442431.3 | -4.12471 | 0.003815 | down |
| MSTRG.557674.6 | -4.13933 | 0.012643 | down |
| MSTRG.1004843.9 | -4.1503 | 0.001982 | down |
| MSTRG.383410.14 | -4.26068 | 0.013148 | down |
| MSTRG.358116.7 | -4.3028 | 0.003 | down |
| MSTRG.572821.1 | -4.30734 | 0.006141 | down |
| MSTRG.108295.6 | -4.3924 | 0.009422 | down |
| MSTRG.762571.34 | -4.49127 | 0.000357 | down |
| MSTRG.358136.1 | -4.5159 | 0.002846 | down |
| MSTRG.47790.91 | -4.56859 | 0.009965 | down |
| MSTRG.47798.1 | -4.5885 | 0.00264 | down |
| MSTRG.358116.3 | -4.63239 | 0.001221 | down |
| MSTRG.357648.2 | -4.79593 | 0.000137 | down |
| MSTRG.160930.1 | -4.83493 | 0.003009 | down |
| MSTRG.1091337.1 | -4.84826 | 0.007555 | down |
| MSTRG.108515.4 | -4.88003 | 0.003453 | down |
| MSTRG.490331.1 | -4.89822 | 0.004712 | down |
| MSTRG.762778.41 | -4.93536 | 0.001712 | down |
| MSTRG.47785.3 | -4.9712 | 0.003073 | down |
| MSTRG.490480.1 | -5.08649 | 0.00356 | down |
| MSTRG.48508.1 | -5.16637 | 0.000684 | down |
| MSTRG.47785.26 | -5.21382 | 0.000774 | down |
| MSTRG.358136.29 | -5.26426 | 0.000344 | down |
| MSTRG.243599.1 | -5.4116 | 0.001939 | down |
| MSTRG.442608.1 | -5.62827 | 0.001016 | down |
| MSTRG.206103.1 | -5.69473 | 0.000725 | down |
| MSTRG.358135.12 | -5.73913 | 0.000131 | down |
| MSTRG.357648.9 | -5.87032 | 7.74E-05 | down |
| MSTRG.572987.4 | -5.96249 | 0.001513 | down |
| MSTRG.442808.1 | -5.97301 | 0.001212 | down |
| MSTRG.762778.6 | -6.24216 | 0.000301 | down |
| MSTRG.901341.1 | -6.4556 | 4.26E-05 | down |
| MSTRG.292667.14 | -7.09657 | 0.001708 | down |
| MSTRG.1032882.4 | -7.33216 | 6.64E-06 | down |
| MSTRG.315532.1 | -7.52674 | 1.80E-05 | down |
| MSTRG.363054.2 | -7.91553 | 6.15E-07 | down |
| MSTRG.571607.3 | -8.40291 | 1.82E-05 | down |
| MSTRG.47785.15 | -9.44314 | 6.61E-08 | down |

FC, fold change.
